# INPP5D/SHIP1-mediated immunometabolic remodeling of renal monocytes in idiopathic membranous nephropathy

**DOI:** 10.3389/fimmu.2026.1755723

**Published:** 2026-03-03

**Authors:** Haoran Dai, Baoli Liu, Hongliang Rui, Liuxiao Yang, Hanxue Jiang, Qihan Zhao, Wu Liu

**Affiliations:** 1 Center of Nephrology & Rheumatology, Beijing Hospital of Traditional Chinese Medicine, Capital Medical University, Beijing, China; 2 Beijing Institute of Chinese Medicine, Beijing, China; 3 School of Traditional Chinese Medicine, Changchun University of Chinese Medicine, Changchun, China

**Keywords:** idiopathic membranous nephropathy, immunometabolism, INPP5D/SHIP1, metabolism, passive Heymann nephritis

## Abstract

**Background:**

Idiopathic membranous nephropathy (IMN) is an antibody-mediated glomerulopathy in which podocyte-directed autoimmunity is well characterized, whereas the immunometabolic programs of innate immune cells within the renal microenvironment remain poorly defined. Src homology-2 domain-containing inositol 5-phosphatase 1 (SHIP1, encoded by *INPP5D*) is a key negative regulator of PI3K signaling in myeloid cells and an emerging immunopharmacologic target, but its role in IMN is unknown.

**Methods:**

Bulk and single-cell RNA-seq analyses were performed using public human IMN datasets, and the passive Heymann nephritis (PHN) rat model was used specifically for *in vivo* validation of key histopathological and signaling readouts to dissect INPP5D/SHIP1-centered immunometabolic pathways in IMN. Public glomerular transcriptomes from IMN and control kidneys were deconvoluted using CIBERSORT and ESTIMATE to quantify immune/stromal components and infer infiltrating leukocyte subsets. Differentially expressed genes were intersected with curated immune- and metabolism-related gene sets to identify immunometabolic hubs. Single-cell RNA-sequencing datasets were used to localize *INPP5D* and related pathways to specific renal cell populations, reconstruct monocyte differentiation trajectories and metabolic states, and infer ligand–receptor communication with podocytes. Key findings were validated in PHN rats by assessing proteinuria, renal histopathology, immune cell markers, podocyte proteins and SHIP1-related signaling molecules.

**Results:**

IMN kidneys exhibited elevated immune and stromal scores, with increased infiltration of monocytes and naïve B cells and a relative depletion of regulatory T cells. Cross-differential analyses identified five overlapping immune–metabolic genes (*INPP5D, PLCG1, KL, ACO1, ARG2*), among which INPP5D was significantly upregulated and predominantly expressed in monocytes. Single-cell analyses revealed that renal monocytes in IMN displayed enhanced steroid biosynthesis, a skewed trajectory toward an M1-like state and strengthened SPP1-mediated communication with podocytes. In PHN rats, we recapitulated key clinical and histological features of IMN, accompanied by increased monocyte/macrophage infiltration, altered podocyte markers, and upregulation of SHIP1 and downstream PI3K/Akt signaling.

**Conclusions:**

These data delineate an INPP5D/SHIP1-centered immunometabolic program in renal monocytes as a potential regulatory factor of pathological monocyte–podocyte crosstalk in IMN. Targeting SHIP1-related PI3K/Akt pathways and monocyte immunometabolism may offer novel immunomodulatory strategies for risk stratification and disease modification in membranous nephropathy.

## 1 Introduction

Idiopathic membranous nephropathy (IMN) is a primary glomerular disease characterized by diffuse thickening of the glomerular basement membrane (GBM) and subepithelial immune complex deposition and represents one of the leading causes of nephrotic syndrome in adults worldwide (1, 2). Despite optimized supportive care, including renin–angiotensin system blockade and strict risk factor control, approximately one-third of patients ultimately progress to end-stage renal disease (ESRD) (1). The identification of podocyte antigens such as M-type phospholipase A2 receptor (PLA2R), thrombospondin type-1 domain-containing 7A (THSD7A) and neural epidermal growth factor-like 1 protein (NELL-1) has firmly established IMN as an organ-specific, antibody-mediated autoimmune disease (1, 3–5). These discoveries have transformed diagnosis and monitoring and have provided a rationale for B-cell–targeted therapies (2, 6–8).

The prevailing pathogenic paradigm emphasizes binding of circulating autoantibodies to podocyte antigens, formation of *in situ* subepithelial immune complexes, complement activation and subsequent podocyte injury, culminating in heavy proteinuria and progressive loss of renal function (5, 7). However, this antibody–complement framework only partially explains the marked heterogeneity in disease course, treatment responses and relapse patterns seen in clinical practice (2). Patients with comparable autoantibody profiles and histological lesions can experience divergent outcomes, and some progress despite apparent immunological remission. These discrepancies suggest that additional layers of regulation within the renal immune microenvironment, beyond the adaptive humoral response, may critically influence IMN pathogenesis and trajectory (9, 10).

Over the past decade, “immunometabolism” has emerged as a key conceptual bridge linking immune cell fate and function to cellular metabolic programs. Activation, differentiation and effector functions of immune cells are tightly coupled to metabolic rewiring, involving coordinated changes in glycolysis, oxidative phosphorylation, amino acid metabolism and lipid and steroid biosynthesis (9–11). Dysregulated immunometabolism has been implicated in a broad spectrum of autoimmune and inflammatory diseases (10, 12). In IMN, however, most mechanistic studies have focused on adaptive immunity—B-cell activation, autoantibody production and helper T-cell imbalances, including Th17/Treg disequilibrium (13–15)—whereas the metabolic states of innate immune cells within the kidney and their contribution to local inflammation and tissue remodeling remain largely unexplored (16, 17).

Monocytes and macrophages are central orchestrators of tissue inflammation and repair and occupy a pivotal position at the interface between innate and adaptive immunity. They can adopt a continuum of activation states, ranging from classically activated, pro-inflammatory M1-like phenotypes to alternatively activated, tissue-repairing M2-like phenotypes (16, 17). These functional states are strongly shaped by metabolic cues and intracellular signaling networks. Among key regulators, Src homology-2 domain-containing inositol 5-phosphatase 1 (SHIP1), encoded by INPP5D, is a critical negative regulator of phosphatidylinositol 3-kinase (PI3K) signaling in myeloid cells (18, 19). SHIP1 integrates signals from cytokine receptors, Fc receptors and pattern recognition receptors and thereby influences immune cell survival, migration and effector functions (19, 20). Importantly, the PI3K/Akt axis sits at the core of immunometabolic regulation, closely linking signal transduction to lipid and steroid metabolism as well as cell survival and activation (21). INPP5D/SHIP1 has attracted growing interest as an immunopharmacologic target in neuroinflammation, viral infection and other inflammatory conditions (22), but its role in IMN has not been defined.

Against this background, we hypothesized that the renal immune microenvironment in IMN is extensively remodeled and that a subset of immunometabolism-related genes, centered on the inositol 5-phosphatase INPP5D/SHIP1, plays a pivotal role in monocyte-driven immunometabolic reprogramming. We further postulated that this INPP5D/SHIP1-anchored program contributes to pathological crosstalk between monocytes and podocytes and may constitute a tractable immunopharmacologic node. To test these hypotheses, we integrated deconvolutional analyses of bulk glomerular transcriptomes with single-cell RNA-sequencing data and an *in vivo* passive Heymann nephritis (PHN) rat model. Our aim was to delineate an INPP5D/SHIP1-centered immunometabolic network in IMN and to generate mechanistic clues that could inform future, target-directed interventions.

## 2 Methods

### 2.1 Public glomerular transcriptomes for immunometabolic profiling in IMN

Gene expression data from renal tissue of patients with IMN and controls were obtained from the Gene Expression Omnibus (GEO) database (accession number GSE108109). Raw microarray data were log_2_-transformed and normalized using the normalizeBetweenArrays function implemented in the limma package (v3.60.3). Probe IDs were mapped to gene symbols based on platform annotation files, and the average expression value was calculated for genes represented by multiple probes. Batch effects were evaluated by principal component analysis (PCA), and when present, were corrected using the removeBatchEffect function in limma. For CIBERSORT(https://cibersortx.stanford.edu/), we used the LM22 signature matrix with 1,000 permutations. Samples with CIBERSORT output P<0.05 were retained, resulting in 44 biopsy-proven IMN cases and 6 control samples for downstream immune proportion analyses.

### 2.2 Deconvolution of the renal immune microenvironment and monocyte infiltration

To characterize immune cell composition in IMN kidneys, we applied the CIBERSORT algorithm to the normalized expression data. Up to 22 immune cell subsets were estimated for each sample and only results with CIBERSORT P < 0.05 were retained for further analysis. The ESTIMATE algorithm was used to calculate immune scores, stromal scores, and ESTIMATE scores, reflecting the overall immune and stromal cell content within the tissue microenvironment. Differences between IMN and control groups were compared using appropriate statistical tests as described below.

### 2.3 Identification of differentially expressed genes and cross-differential immunometabolic genes

Differentially expressed genes (DEGs) between IMN and control samples were identified using the limma package in R. Genes with an adjusted P-value < 0.05 and |logFC| ≥ 0.5 were considered significantly differentially expressed. To identify potential immunometabolic hub genes, we intersected DEGs with curated immune-related and metabolism-related gene sets from public resources.

Immune-related genes were collected from ImmPort (https://www.immport.org/home). Metabolism-related genes were collected from Reactome (https://reactome.org). The overlapping genes were defined as cross-differential immunometabolic genes and were subjected to further functional enrichment and network analyses.

### 2.4 Functional enrichment analysis

Gene Ontology (GO) and Kyoto Encyclopedia of Genes and Genomes (KEGG) pathway enrichment analyses were performed for DEGs and cross-differential immunometabolic genes using the clusterProfiler package in R. Biological processes, molecular functions, cellular components, and signaling pathways with adjusted P-values < 0.05 were considered significantly enriched. The results were visualized using bar plots and bubble plots to highlight key pathways potentially involved in IMN pathogenesis.

### 2.5 Single-cell data analysis of IMN

To validate and refine the findings at single-cell resolution, we analyzed publicly available single-cell RNA sequencing datasets (GSE171458) of human kidney tissue that included 6 IMN cases and 2 control samples. The “Seurat” package was used for single-cell analysis, including cell type identification and clustering. The “scMetabolism” package was employed to identify differential metabolic pathways in the single-cell data. The “Monocle 3” package was used for single-cell trajectory analysis, and the “CellChat” package was used for analysis of cell-to-cell interactions.

### 2.6 Experimental animals and PHN model

Six-week-old male specific pathogen-free (SPF) Sprague–Dawley rats (160–180 g) were purchased from a certified laboratory animal supplier. All animals were housed under controlled conditions (12-h light/dark cycle, temperature 22 ± 2°C, humidity 55 ± 5%) with free access to food and water. After one week of acclimatization, rats were randomly assigned to control and PHN groups, with 5 rats in each group. Control group (CON) received 0.5 mL/100g normal saline via tail vein injection (23). PHN group were injected with 0.5 mL/100g sheep anti-rat Fx1A antibody serum (PTX-002S, Probetex, USA) via tail vein. One-week post-injection, 24-hour urinary protein (24hUTP) levels were measured. Successful modeling was assessed using a composite panel including: (1) increased 24-hour urinary protein excretion (>10mg), (2) reduced serum albumin, and (3) characteristic glomerular lesions on light microscopy and electron microscopy. After 12 weeks, the rats were anesthetized with 1% pentobarbital sodium, and blood was drawn from the abdominal aorta. Kidney samples were also collected for subsequent analysis. Every animal experiment was approved by the Animal Committee of Beijing Hospital of Traditional Chinese Medicine Affiliated to Capital Medical University (ethics number BJTCM-R-2024-07-02) and was conducted in accordance with the ARRIVE guidelines related to animal handling.

### 2.7 Biochemical measurements

Urine was collected from rats every two weeks using metabolic cages for 24-hour periods. The collected abdominal aorta blood was centrifuged to obtain the serum (at 3000 rpm, 4 °C, 10 min). Total urinary protein levels were measured using the CBB method (C035-2-1, Nanjing Institute of Jiancheng Bioengineering, China). Albumin were measured using automatic biochemical analyzer.

### 2.8 Renal histopathology and transmission electron microscopy

At the end of the experimental period, rats were anesthetized, and kidneys were harvested. Renal tissue was fixed in 4% paraformaldehyde, embedded in paraffin, and sectioned at 3 µm thickness. Sections were stained with hematoxylin and eosin (HE), Masson, and periodic acid–silver methenamine (PASM)-Masson to evaluate glomerular structure, mesangial expansion, and GBM thickening. Histological changes were examined under light microscopy, and semi-quantitative scoring was performed by a blinded pathologist. For ultrastructural examination, small pieces of cortex were fixed in glutaraldehyde, post-fixed in osmium tetroxide, dehydrated, embedded in resin, and cut into ultrathin sections. Sections were stained with uranyl acetate and lead citrate and examined by transmission electron microscopy (TEM) to assess subepithelial electron-dense deposits and podocyte foot process effacement.

### 2.9 Western blot analysis

Total protein was extracted from renal cortex using RIPA lysis buffer containing protease and phosphatase inhibitors. Protein concentrations were determined using a BCA assay. Equal amounts of protein were separated by SDS-PAGE and transferred onto PVDF membranes. After blocking, membranes were incubated overnight at 4°C with primary antibodies against PI3K (CST, 4292, US), p-PI3K (SAB, 12057, China), Akt (Abcam, Ab179463, UK), p-Akt (CST, 4060, US), nephrin (Abcam, Ab216341, UK), synaptopodin (Abcam, Ab259976, UK), iNOS (Proteintech, 18985-1-AP, China), ARG1 (Proteintech, 16001-1-AP, China), and GAPDH (Proteintech, 60004-1-Ig, China). After incubation with appropriate HRP-conjugated secondary antibodies, bands were visualized using enhanced chemiluminescence and quantified by ImageJ software. Protein expression levels were normalized to GAPDH.

### 2.10 Immunofluorescence and immunohistochemistry staining

Paraffin-embedded kidney sections were deparaffinized, rehydrated, and subjected to antigen retrieval. After blocking with normal serum, sections were incubated with primary antibodies against CD163 (Abmart, TD8235S, China) and synaptopodin (Santa Cruz, sc-515842, US) followed by fluorescently labeled secondary antibodies. Nuclei were counterstained with DAPI. Immunohistochemistry was performed for nephrin (Abcam, Ab216341, UK), SHIP1 (Immunoway, YT4290, China), and CD86 (proteintech, 13395-1-AP, China), and then treated with corresponding secondary antibodies, and stained with DAB. Finally, quantitative analysis of the positive staining in the glomeruli was conducted using ImageJ. The integrated optical density (IOD) was used as a quantitative standard.

### 2.11 Dual immunofluorescence staining with TSA amplification

The renal sections (3 µm) were stained with dual immunofluorescence using the TSA method. The sections were sequentially incubated with primary antibodies against SHIP1 and iNOS, and simultaneously treated with HRP-conjugated secondary antibody and TSA for signal amplification. The nuclear dots were stained with DAPI for comparison.

### 2.12 Statistical analysis

Statistical analysis was performed using IBM SPSS Statistics 27.0 software. Data are presented as mean ± standard deviation. Independent T-tests were used to compare differences between groups. A P-value < 0.05 was considered statistically significant.

## 3 Results

### 3.1 Remodeling of the renal immune microenvironment with enrichment of monocyte immunometabolism in IMN

**Figure 1:**
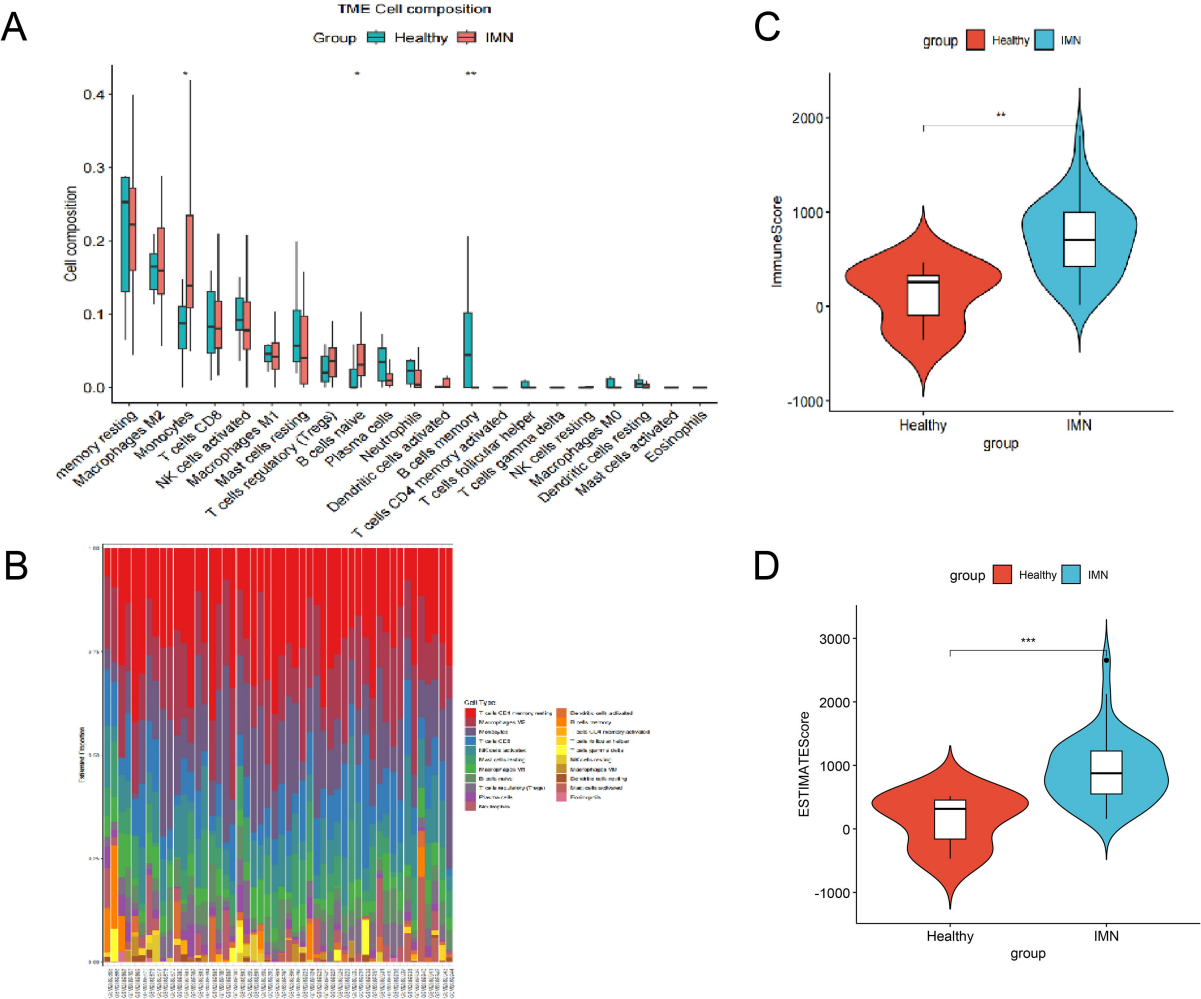
Remodeling of the renal immune microenvironment and enrichment of monocytes in IMN kidneys. **(A)** Histogram showing the relative proportions of 22 immune cell subsets in kidney tissue samples from GSE108109, highlighting increased monocyte infiltration in IMN compared with healthy controls (Kruskal–Wallis test). **(B)** Stacked bar plot of immune cell composition in individual samples. **(C)** Comparison of immune scores between IMN and healthy controls, indicating an immune-activated microenvironment. **(D)** Comparison of ESTIMATE scores between IMN and healthy controls, reflecting combined immune and stromal remodeling in IMN kidneys. *P<0.05; **P<0.01; ***P<0.001.

To systematically characterize immune dysregulation in IMN, we first applied CIBERSORT and ESTIMATE to the bulk transcriptomic data. As shown in **Figures 1A–D**, Compared with controls, IMN kidney samples exhibited significantly higher immune scores, stromal scores, and ESTIMATE scores, indicating increased immune cell infiltration and stromal remodeling. CIBERSORT deconvolution revealed distinct alterations in immune cell composition: IMN samples showed an increased proportion of monocytes and naïve B cells, accompanied by a decrease in memory B cells. These findings suggest a shift from a stable memory B-cell pool toward a continuously replenished naïve/activated compartment, consistent with ongoing antigenic stimulation and active humoral immune responses. Together, these results indicate that IMN kidneys are not immunologically quiescent but instead display a substantially remodeled immune microenvironment.

### 3.2 Cross-differential immunometabolic genes highlight INPP5D/SHIP1 as a monocyte-centered hub

**Figure 2:**
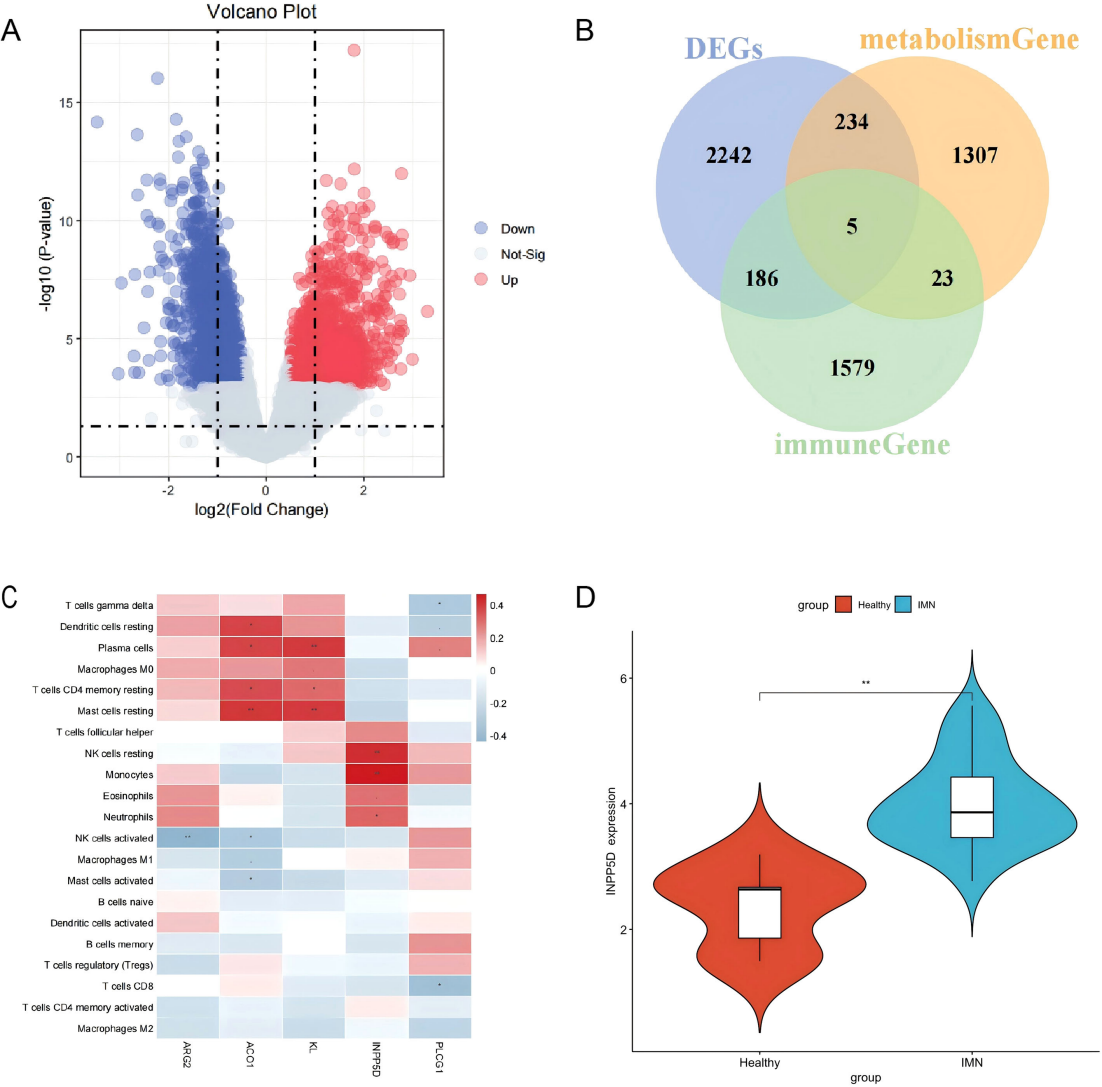
Cross-differential immunometabolic genes highlight INPP5D/SHIP1 as a monocyte-centered hub in IMN. **(A)** Volcano plot of differentially expressed genes (DEGs) between IMN and healthy controls in GSE108109. **(B)** Venn diagram showing the intersection of IMN DEGs with immune-related and metabolism-related gene sets, identifying five cross-differential immunometabolic genes *(INPP5D, PLCG1, KL, ACO1, ARG2*). **(C)** Correlation heatmap between immune cell abundance and expression of the five cross-differential genes, emphasizing the positive association between INPP5D/SHIP1 expression and monocyte infiltration. **(D)** Boxplot comparing INPP5D expression between IMN and healthy subjects, demonstrating significant upregulation of INPP5D/SHIP1 in IMN kidneys.

As shown in **Figure 2A**, to explore molecular hubs connecting immune dysregulation with metabolic remodeling, we identified DEGs between IMN and control samples and intersected them with curated immune-related and metabolism-related gene sets. This analysis yielded five cross-differential immunometabolic genes: *INPP5D, PLCG1, KL, ACO1*, and *ARG2* (**Figure 2B**, **Table 1**). Among these candidate genes, *INPP5D* (encoding SH2 domain inositol 5-phosphatase-1, also known as SHIP1) stands out particularly. Correlation analysis revealed that the expression level of *INPP5D* was significantly positively correlated with the abundance of monocytes in renal tissue (**Figure 2C**). More importantly, compared with the healthy control group, the expression of *INPP5D* was significantly increased in IMN patients (P < 0.05) (**Figure 2D**). The convergence of this series of evidence is highly enlightening. First, there is an increase in the number of monocytes in the kidneys; second, INPP5D is a key immune-metabolic regulatory gene; finally, the expression of *INPP5D* precisely synchronizes with the increased number of monocytes. Collectively, these convergent observations nominate INPP5D/SHIP1 as a candidate immunometabolic hub linked to monocyte-rich IMN microenvironments.

**Table 1:** The characteristics of 5 DEGs in the intersection of immunity and metabolism.

Gene symbol	Protein name	Core functional annotation	P-value
*INPP5D*	SHIP1	Phosphatidylinositol-3,4,5-trisphosphate 5-phosphatase 1, negatively regulates the PI3K signaling pathway	< 0.05
*PLCG1*	PLC-γ1	Phospholipase C-γ1, involved in intracellular signal transduction	< 0.05
*KL*	Klotho	α-Klotho protein, anti-aging, regulates ion channels	< 0.05
*ACO1*	IRP1	Iron regulatory protein 1/Cytoplasmic aconitase, involved in iron metabolism and energy metabolism	< 0.05
*ARG2*	Arginase-2	Arginase-2, involved in the urea cycle and amino acid metabolism	< 0.05

### 3.3 Single-cell mapping of INPP5D/SHIP1-high monocytes and steroid biosynthesis programs

**Figure 3:**
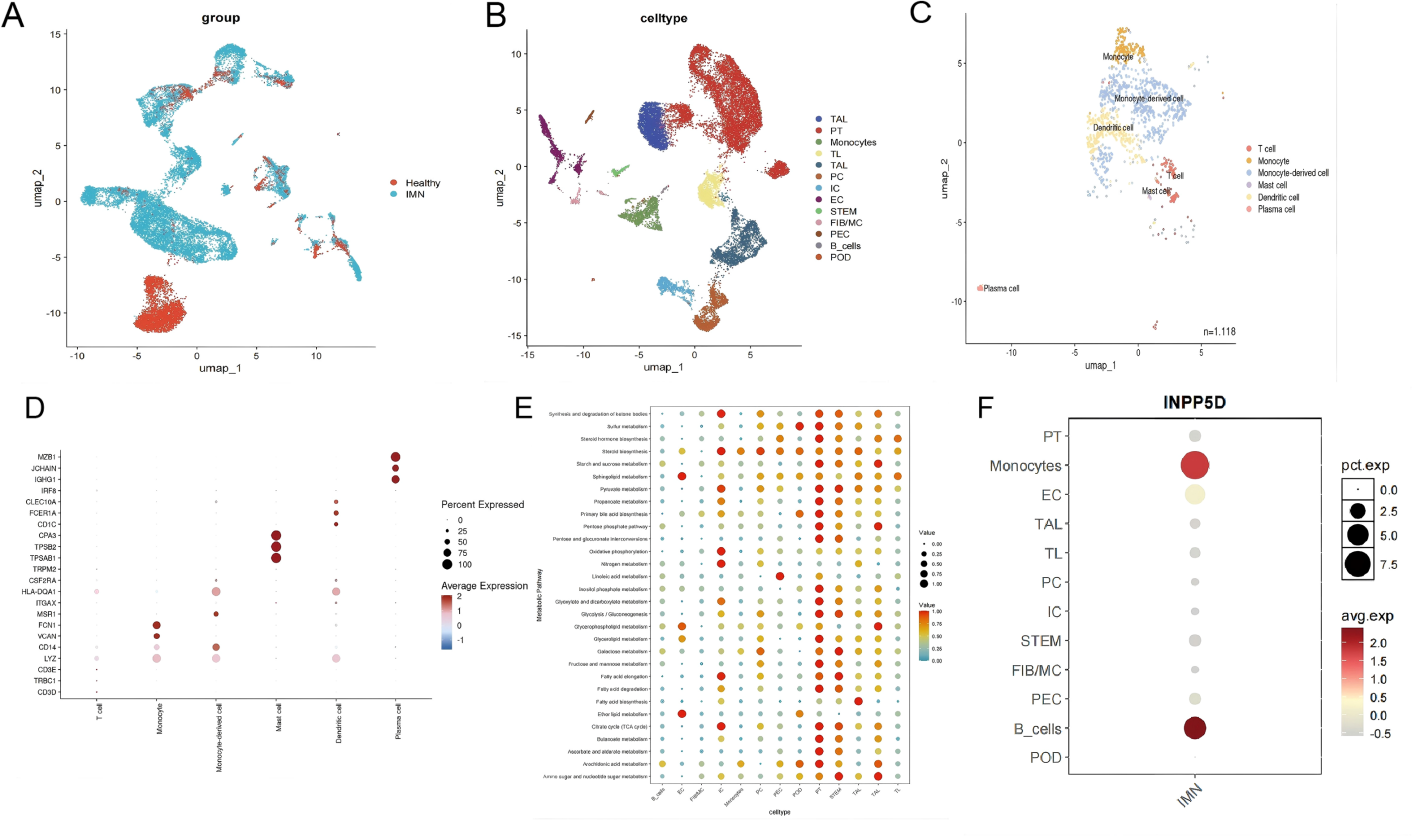
Single-cell mapping of INPP5D/SHIP1-high monocytes and their immunometabolic reprogramming in IMN. **(A)** UMAP plot showing the distribution of cells from IMN and healthy control kidneys in the single-cell RNA-seq dataset. **(B)** UMAP plot identifying 13 major renal cell clusters. **(C)** UMAP plot highlights immune cell subpopulations, including monocytes. **(D)** Heatmap of marker genes used to define immune cell subsets. **(E)** Metabolic pathway activity across cell subpopulations, demonstrating selective activation of the steroid biosynthesis pathway in IMN monocytes. **(F)** Feature plot of *INPP5D* expression, showing predominant INPP5D/SHIP1 expression in monocyte clusters compared with other renal cell types. pct.exp, percentage expression; avg.exp, average expression.

To validate the above findings at single-cell resolution and identify specific cell populations harboring INPP5D expression, we analyzed single-cell RNA-seq data from human kidney tissue. Clustering and cell type annotation revealed major parenchymal cells, including proximal tubular cells, distal tubular cells, collecting duct cells, endothelial cells, and podocytes, as well as multiple immune cell subsets (**Figures 3A, B**). We reclustered the immune cells in the kidneys (**Figure 3C**), using the marker genes as shown in **Figure 3D**. INPP5D expression was predominantly localized to monocyte clusters, with much lower expression in other cell types (**Figure 3F**).

Evaluating the metabolic pathway activity of each cell subpopulation, we identified a key metabolic event. Compared to healthy controls, the steroid biosynthesis pathway in monocytes of IMN patients was significantly activated (**Figure 3E**). This finding suggests that IMN-associated monocytes undergo specific metabolic reprogramming toward steroid biosynthesis, potentially enabling them to modulate local immune responses and the microenvironment in a distinct manner.

### 3.4 Monocyte immunometabolic trajectory and monocyte–podocyte crosstalk in IMN

**Figure 4:**
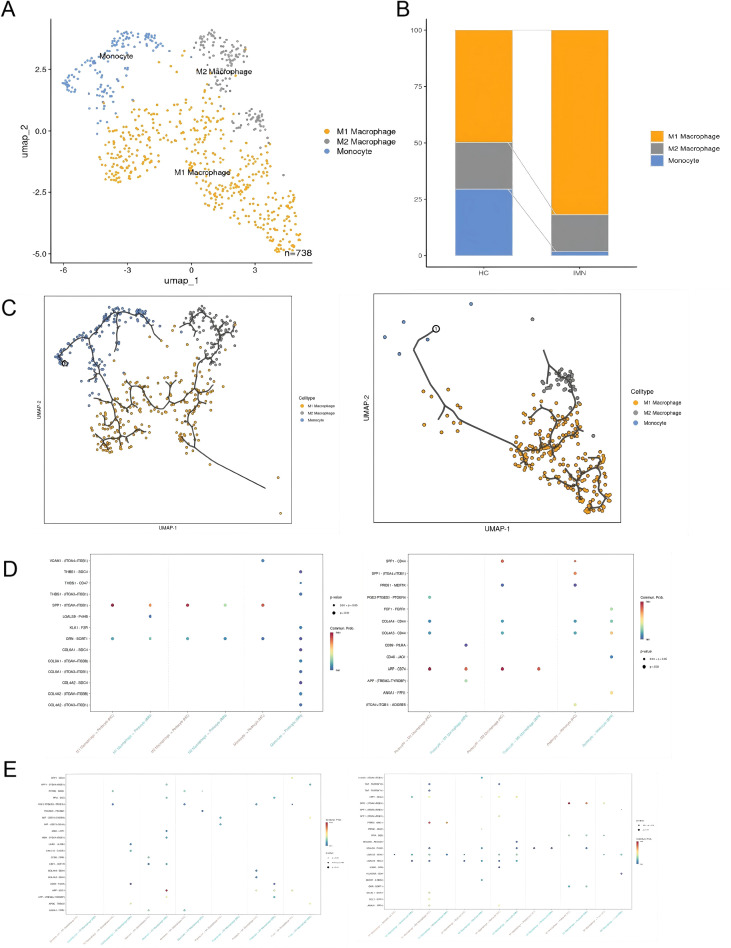
Monocyte immunometabolic trajectory and SPP1-mediated monocyte–podocyte communication in IMN. **(A)** UMAP plot showing monocyte subclusters in renal tissue. **(B)** Bar plot comparing the proportions of monocyte subclusters between healthy controls and IMN, indicating expansion of specific monocyte subsets in IMN. **(C)** Pseudotime trajectory analysis of monocytes in healthy controls (left) and IMN (right), revealing a shift toward an M1-like, pro-inflammatory state in IMN. **(D)** CellChat-derived network of ligand–receptor interactions between monocytes/monocyte subsets and podocytes, highlighting strengthened SPP1 (osteopontin) signaling from INPP5D/SHIP1-high monocytes to podocytes. **(E)** Ligand–receptor interaction network between monocytes/monocyte subsets and other immune cells, illustrating broader immunometabolic crosstalk within the IMN renal microenvironment.

We conducted a subgroup analysis of monocytes in the IMN, and found that the proportion of M1 macrophages increased in the MN (**Figures 4A, B**). We next performed trajectory analysis using Monocle 3 to explore the dynamic changes and differentiation states of monocytes in IMN. Compared with controls, monocytes from IMN kidneys followed a distinct pseudotime trajectory biased toward an M1-like, pro-inflammatory state, indicating that their activation landscape is reshaped within the diseased microenvironment (**Figure 4C**).

To understand how these monocytes interact with neighboring cells, we used CellChat to infer cell–cell communication networks based on ligand–receptor expression patterns. This analysis revealed intensified crosstalk between monocytes and podocytes in IMN, with the SPP1 (osteopontin) signaling axis emerging as a key pathway (**Figures 4D, E**). Monocytes in IMN exhibited increased SPP1 expression, while podocytes expressed corresponding receptors, suggesting that osteopontin-mediated signaling may contribute to cytoskeletal remodeling, adhesion changes, and injury in podocytes. These results suggest that the monocyte subpopulation with high INPP5D expression may conduct signal transmission with podocytes through the SPP1 axis, and the SPP1 axis as a possible medium for monocyte-podocyte communication is worthy of direct functional testing.

### 3.5 PHN rats recapitulate the INPP5D/SHIP1-related immunometabolic injury pattern of IMN

**Figure 5:**
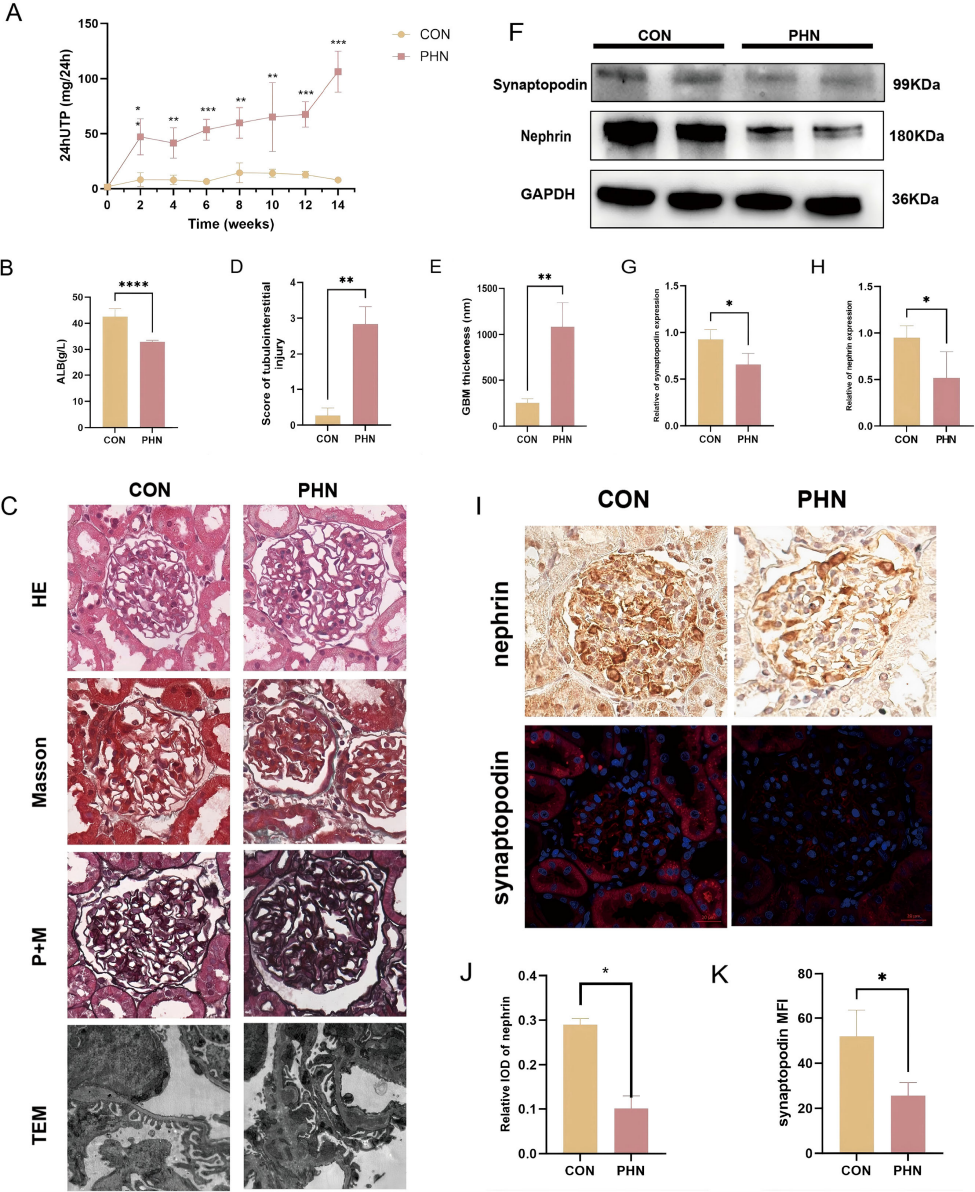
Assessment of renal injury and podocyte markers in PHN rats. **(A)** Time course of 24-h urinary total protein excretion in control and PHN rats. **(B)** Comparison of serum albumin levels between the two groups. **(C)** Representative light microscopy images (×400, scale bar = 20 μm) and transmission electron microscopy images (×10,000) of glomerular lesions. **(D)** Semiquantitative scoring of tubulointerstitial injury. **(E)** Quantification of GBM thickness by electron microscopy in the two groups. **(F)** Western blot analysis of the podocyte marker proteins nephrin and synaptopodin. **(G, H)** Densitometric analysis of nephrin and synaptopodin expression in control and PHN rats. **(I)** Representative immunohistochemistry and immunofluorescence images of glomerular nephrin and synaptopodin expression (×400, scale bar = 20 μm). **(J, K)** Quantification of nephrin and synaptopodin staining intensity in the two groups of rats. *P<0.05; **P<0.01; ***P<0.001; ****P<0.0001.

To validate our bioinformatics findings *in vivo*, we established a PHN rat model. **Figure 5** showed the construction of the PHN rat model. The rats developed progressive proteinuria and a decrease in ALB compared with controls. Light microscopy revealed diffuse GBM thickening and “spike” formation, along with varying degrees of mesangial expansion, closely resembling the histological features of human IMN, suggesting the success of the model establishment. TEM demonstrated characteristic subepithelial electron-dense deposits and widespread podocyte foot process effacement. Immunofluorescence staining showed reduced expression of podocyte slit diaphragm proteins nephrin and synaptopodin in PHN glomeruli. These findings confirm that the PHN model faithfully reproduces key clinical and pathological hallmarks of IMN and provides a suitable platform for mechanistic investigation.

### 3.6 SHIP1-related signaling, M1-biased macrophage activation and podocyte injury in PHN kidneys

**Figure 6:**
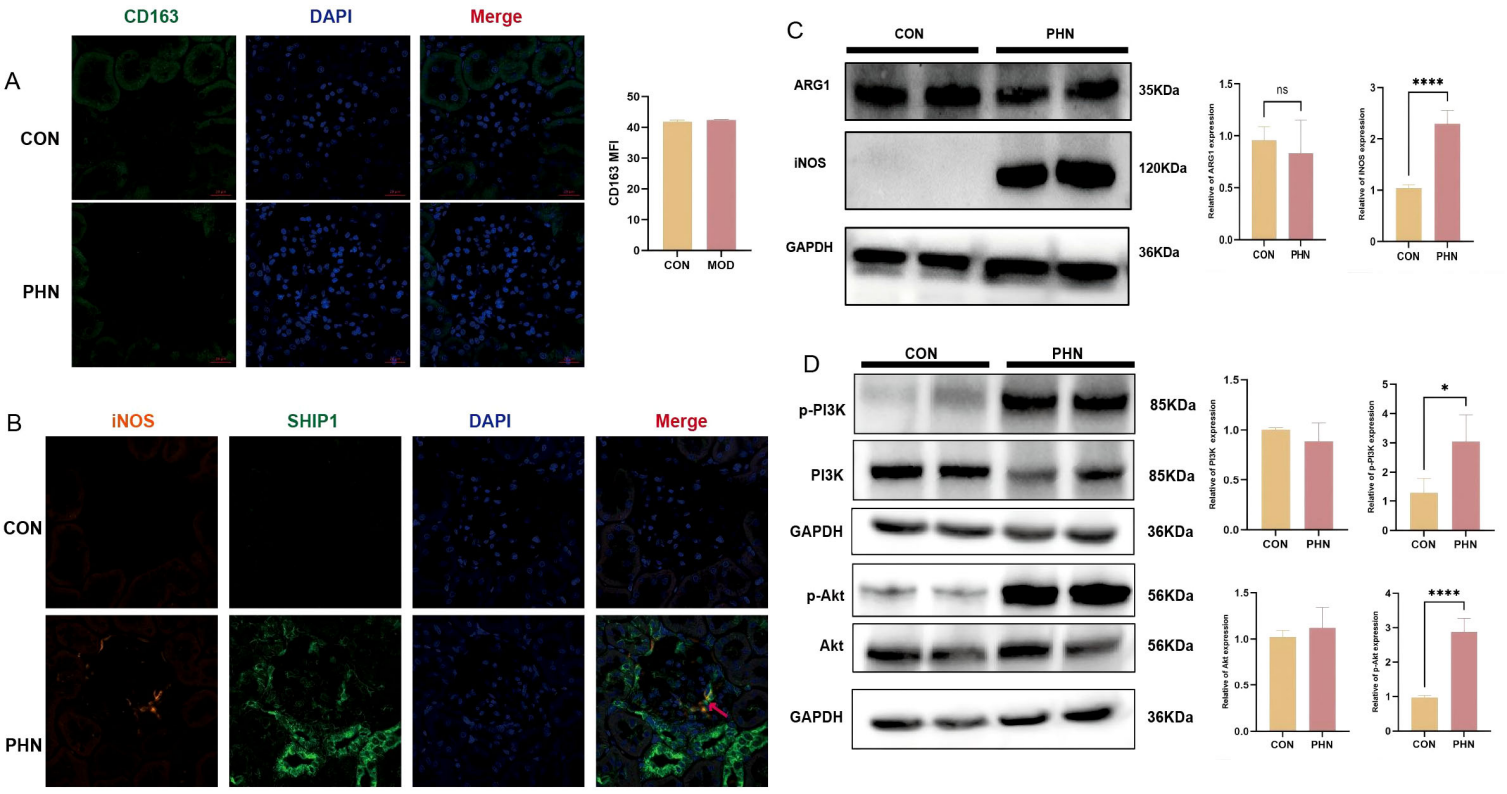
SHIP1-related macrophage polarization and downstream signaling in PHN kidneys. **(A)** Representative immunofluorescence images showing M2 (CD163) macrophage marker proteins in rat glomeruli. **(B)** Immunofluorescence co-staining revealed that the M1 marker iNOS and SHIP1 were co-localized (indicated by the red arrow). **(C)** Western blot analysis of M1- and M2-associated macrophage marker proteins in renal tissue. **(D)** Western blot analysis of downstream components of SHIP1 signaling, including PI3K, AKT and their phosphorylated forms. *P<0.05; ****P<0.0001. ns, no statistical significance.

Immunofluorescence did not detect the infiltration of M2 marker CD163 between the two groups (**Figure 6A**). However, through immunofluorescence co-staining, a high expression level of iNOS was observed, and it was found to be co-localized with SHIP1 (**Figure 6B**). At the polarization markers, the expression of iNOS was significantly upregulated, while the expression of ARG1 did not show a significant increase (**Figure 6C**). This indicates that M1-type macrophages are dominant. Western blot analysis also showed that, compared with the control group, the downstream proteins PI3K and Akt of SHIP1 in PHN kidneys were also in an activated state (**Figure 6D**). Collectively, these data indicate that PHN is accompanied by M1-biased macrophage activation, increased SHIP1 expression, enhanced PI3K/Akt pathway phosphorylation, and loss of podocyte structural proteins. These findings support the involvement of the INPP5D/SHIP1-related signaling network in the body. However, they do not align with the co-upregulation of SHIP1 and PI3K/AKT as well as the biochemical conditions. The directionality and cell type specificity of this SHIP1 - PI3K/Akt regulatory mechanism require further mechanistic analysis.

## 4 Discussion

Idiopathic membranous nephropathy has classically been viewed as an organ-specific, antibody-mediated autoimmune disease in which circulates autoantibodies against podocyte antigens such as PLA2R, THSD7A and NELL-1 form subepithelial immune complexes, activate complement and injure podocytes and the glomerular filtration barrier (3–6, 8). This framework, although highly successful in explaining antigen specificity and guiding B-cell–directed therapy, is largely centered on adaptive immunity and humoral responses and only partially accounts for the marked heterogeneity in clinical course and treatment responses (2, 7). It also leaves the contribution of innate immune cells and their immunometabolic states within the kidney microenvironment insufficiently explored (13, 14).

In this study, we re-examined IMN from an integrated immune–metabolic perspective with a specific focus on the inositol 5-phosphatase INPP5D/SHIP1, an emerging immunopharmacologic target (18, 19). By combining deconvolutional bulk transcriptomics, single-cell RNA-sequencing and an established PHN rat model, we delineated an INPP5D/SHIP1-centered immunometabolic program in renal monocytes. IMN kidneys exhibited a profoundly remodeled immune landscape, with increased immune and stromal scores and enhanced infiltration of monocytes alongside naïve B cells, rather than an immunologically quiescent biopsy picture. Within this milieu, *INPP5D* emerged as a potential key immune–metabolic hub, predominantly expressed in monocytes and linked to a shift toward an M1-like trajectory, augmented steroid biosynthesis and strengthened SPP1-mediated monocyte–podocyte communication. In PHN rats, we recapitulated key clinicopathological features of IMN and observed upregulation of SHIP1 together with activation of downstream PI3K/Akt signaling, supporting engagement of this axis *in vivo* during antibody-mediated podocyte injury.

By deconvoluting bulk transcriptomic data from IMN renal tissue, we first demonstrated a markedly remodeled immune landscape. Compared with healthy controls, IMN kidneys showed higher immune and ESTIMATE scores, indicating increased immune cell infiltration and stromal remodeling. Within this altered cellular composition, we observed an increased proportion of monocytes and naïve B cells, accompanied by a reduction in memory B cells, suggesting a shift toward a continuously replenished naïve/activated compartment in line with persistent antigenic stimulation. At the same time, the expansion of monocytes points to enhanced innate immune activation, which has been relatively under-appreciated in IMN compared with Th17/Treg disequilibrium and B-cell dysregulation.

To search for molecular hubs linking immune dysregulation with metabolic remodeling, we intersected IMN-related DEGs with curated immune- and metabolism-related gene sets and identified five candidates (*INPP5D, PLCG1, KL, ACO1, ARG2*). Among these, *INPP5D* (encoding SHIP1) stood out because it was significantly upregulated in IMN versus controls, its expression correlated positively with monocyte abundance, and scRNA-seq localized its expression predominantly to monocyte clusters. These converging lines of evidence collectively elevate *INPP5D* may serve as a biologically rational immune-metabolic hub in IMN monocytes.

Single-cell metabolic pathway analysis provided additional functional context. In IMN kidneys, monocytes exhibited pronounced activation of the steroid biosynthesis pathway compared with controls, whereas other major immune or parenchymal cell types did not show the same pattern. Steroids are potent immunomodulators, and their local synthesis by myeloid cells is increasingly recognized as an important layer of immune regulation (24, 25). This study revealed a significant activation of the steroid biosynthesis pathway in renal monocytes of IMN patients. This finding provides a new perspective for understanding the heterogeneity of clinical treatment for IMN. In clinical practice, although glucocorticoids are the core drug for inducing remission of IMN, there are significant differences in the response to hormone therapy among different patients, with some patients developing resistance or dependence on steroids. Our data indicate that the local steroid synthesis in monocytes may form an “endogenous regulatory buffer zone” in the glomerular microenvironment. This local steroid metabolic activity may interfere with the pharmacological effects of exogenous corticosteroids by altering the activation threshold of monocytes themselves or regulating the hormone receptor sensitivity of adjacent podocytes.

Furthermore, we observed an enhancement of the SHIP1-PI3K/AKT signaling axis. SHIP1 is a negative regulator of the PI3K signaling pathway, while the PI3K/Akt axis integrates metabolic and survival signals. However, in this study, we found that the expression levels of both were simultaneously increased. Therefore, it can be reasonably assumed that, first, the simultaneous upregulation of SHIP1 and p-AKT may reflect the “compensatory negative feedback mechanism failure” commonly seen in the oligo-inflammatory state of MN. To clarify this, we also conducted a co-localization experiment of SHIP1 and iNOS, observing that the upregulation of SHIP1 was mainly concentrated in the infiltrating M1-type macrophages in the renal glomeruli. Although the cells attempted to inhibit the overactive PI3K pathway by inducing SHIP1, this endogenous response was insufficient to overcome the strong upstream immune complexes and complement stimulation. Additionally, the product of SHIP1, PI(3,4)P2, has also been reported to recruit and activate Akt under certain conditions, which explains why although SHIP1 increased, p-Akt remained at a relatively high level. Secondly, the phosphorylation indicators we measured were conducted throughout the entire kidney tissue, where p-Akt can reflect the combined contribution of various cell types (immune cells and renal parenchymal cells), and thus may not be one-to-one mapped to the intrinsic SHIP1 function of monocytes. Thirdly, the function of SHIP1 is influenced by post-translational regulation and subcellular localization, which can regulate the downstream signaling results. In summary, our data support the involvement of the SHIP1-PI3K/Akt network in the antibody-mediated podocyte injury process, but these data do not allow for a simple linear interpretation and still warrant further in-depth exploration.

Trajectory analysis further revealed that monocytes in IMN follow a distinct differentiation path compared with those in healthy kidneys, with skewing toward an M1-like macrophage state. This is consistent with our *in vivo* data in PHN rats, in which iNOS expression was upregulated while ARG1 levels remained unchanged, indicating a predominance of M1-type macrophages. The apparent tension between these findings and the IgG4-dominant, relatively complement-sparing immune profile traditionally associated with PLA2R-positive IMN deserves attention (1, 3). Our data supports a model in which, despite a Th2/IgG4-biased autoantibody response, the local innate compartment is not uniformly anti-inflammatory; instead, M1-polarized monocytes/macrophages can coexist and actively contribute to podocyte injury, especially in phases of disease activation or relapse.

Cell–cell interaction analysis using CellChat showed that IMN monocyte subsets, particularly M1-like cells, engage in intensified ligand–receptor communication with podocytes and other immune cells. The SPP1 (osteopontin) axis emerged as a prominent signal between monocytes and podocytes (26, 27). Osteopontin is a multifunctional cytokine/adhesion molecule that signals through integrins and CD44 to regulate cytoskeletal dynamics, adhesion and survival (28, 29). Enhanced SPP1-mediated signaling from activated monocytes may therefore promote cytoskeletal disorganization, detachment and apoptosis of podocytes, amplifying proteinuria and glomerular damage. This is in line with the reduction of podocyte markers (nephrin, synaptopodin) and the ultrastructural changes (subepithelial deposits, GBM thickening, foot process effacement) we observed in PHN rats.

The PHN model used in this study successfully reproduced key clinical, pathological and molecular features of human IMN, including progressive proteinuria, GBM thickening with spike formation, subepithelial electron-dense deposits and loss of podocyte slit diaphragm proteins. Importantly, PHN kidneys also displayed upregulated SHIP1 and activation of downstream PI3K and Akt, implying that the INPP5D/SHIP1–PI3K/Akt axis is engaged *in vivo* during antibody-mediated podocyte injury.

Taken together, our findings support a working model in which INPP5D/SHIP1-high monocytes undergo immunometabolic reprogramming toward enhanced steroid biosynthesis and an M1-biased activation state and engage in intensified osteopontin-mediated crosstalk with podocytes. In this model, monocytes not only act as passive responders to immune complexes but also as active coordinators of the glomerular microenvironment, amplifying and maintaining podocyte injury. This represents another possible pathogenic mechanism besides antibody-complement-mediated disease. This concept broadens the current view of IMN pathogenesis and highlights the innate immune compartment as an active and potentially targetable participant in disease progression.

INPP5D/SHIP1 and its downstream network may have dual utility as biomarkers and therapeutic targets. Small-molecule SHIP1 modulators and IL-10–SHIP1 signaling have shown anti-inflammatory efficacy in other settings, suggesting a potential avenue for re-balancing monocyte responses in IMN (22). At the same time, SHIP1 exerts complex, context-dependent roles across immune subsets, acting as both inhibitor and facilitator under distinct conditions, so indiscriminate systemic modulation may be hazardous. Future pharmacologic strategies will likely require cell- and tissue-specific approaches, such as monocyte-targeted delivery, ex vivo reprogramming or combination regimens that fine-tune SHIP1 activity within a defined therapeutic window.

This study has several limitations. First, the human transcriptomic analyses relied on publicly available datasets with relatively small sample sizes, the control group size in GSE108109 was limited (n=6), which may reduce the stability of deconvolution and DEG results. In the future, we should verify this result through a large-sample cohort study, and potential selection bias, batch effects and platform differences cannot be fully excluded despite rigorous preprocessing. Second, CIBERSORT- and ssGSEA-based estimates provide inferred rather than directly measured immune cell proportions and pathway activities; their accuracy in kidney tissue, where immune and non-immune cells are tightly intermingled, requires cautious interpretation. Third, the single-cell dataset included a limited number of IMN and control samples and may not fully capture inter-patient heterogeneity across autoantigen subtypes and disease stages. Fourth, our *in vivo* validation used a modest number of PHN rats and did not incorporate genetic or pharmacologic manipulation of INPP5D/SHIP1, so the causal role of SHIP1 in mediating monocyte immunometabolic changes and podocyte injury remains inferential rather than formally demonstrated.

Future work should extend these observations in several directions. Larger, well-phenotyped IMN cohorts integrating bulk and single-cell omics with longitudinal clinical outcomes are needed to validate INPP5D/SHIP1-related signatures as prognostic markers and to link monocyte immunometabolic states to treatment responses. Mechanistic studies employ monocyte-specific *INPP5D* knockdown/overexpression, conditional knockout models in PHN or other IMN-relevant systems and direct assessments of steroid biosynthesis and osteopontin production by monocytes/macrophages and their impact on podocyte cytoskeleton and survival will be essential to establish causality. Finally, preclinical evaluation of SHIP1-targeted or SPP1-blocking interventions, ideally using monocyte-focused delivery strategies, may help define the therapeutic window in which modulation of this pathway can achieve disease-modifying effects in membranous nephropathy without incurring unacceptable off-target immune perturbations.

## 5 Conclusion

In conclusion, multi-layer transcriptomic analyses (bulk deconvolution and single-cell RNA-seq) were performed using public human IMN datasets, and the PHN rat model was used specifically for *in vivo* validation of key histopathological and signaling readouts. we delineated an INPP5D/SHIP1-centered immunometabolic program in IMN. *INPP5D*-high monocytes exhibit M1-like skewing, enhanced steroid biosynthesis, and intensified SPP1-mediated podocyte crosstalk via the PI3K/Akt axis. These findings extend the classical antibody-mediated paradigm by highlighting the active role of innate immunometabolism in disease progression. Consequently, the INPP5D/SHIP1 axis emerges as a promising biomarker and therapeutic target, providing a rationale for monocyte-focused strategies—such as SHIP1 modulation or SPP1 blockade—to refine risk stratification and treatment in membranous nephropathy.

## Data Availability

Publicly available datasets were analyzed in this study. This data can be found here: GSE108109,GSE171458.
